# P-413. Epidemiology of *Clostridioides difficile* and Bloodstream Infection in Belgium: Insights from 2014-2022 Data

**DOI:** 10.1093/ofid/ofae631.614

**Published:** 2025-01-29

**Authors:** Nathalie Shodu, Milena Callies, Louise Vaes, Karl Mertens, Boudewijn Catry

**Affiliations:** Sciensano, Brussels, Brussels Hoofdstedelijk Gewest, Belgium; Sciensano, Brussels, Brussels Hoofdstedelijk Gewest, Belgium; Sciensano, Brussels, Brussels Hoofdstedelijk Gewest, Belgium; Sciensano, Brussels, Brussels Hoofdstedelijk Gewest, Belgium; Sciensano, Brussels, Brussels Hoofdstedelijk Gewest, Belgium

## Abstract

**Background:**

Healthcare-associated infections (HAI), like bloodstream infections (BSI) and Clostridioides difficile (CDI), strain Belgian hospitals. Since 2014, national surveillance requires data on hospital-associated (HABSI) and central line-associated bloodstream infections (CLABSI). Although CDI surveillance was obligatory until 2014, hospitals must join a surveillance program since 2015. This study delves into BSI and CDI trends in Belgium from 2014 to 2022.

Mean incidence of central line-associated bloodstream infections,hospital-wide ans in intensive care, Belgium 2013-2022, (CLABSI,central line-associated bloodstream infections; CRBSI, catheter related bloodstream infection=Confirmed CLABSI)

**Figure:**
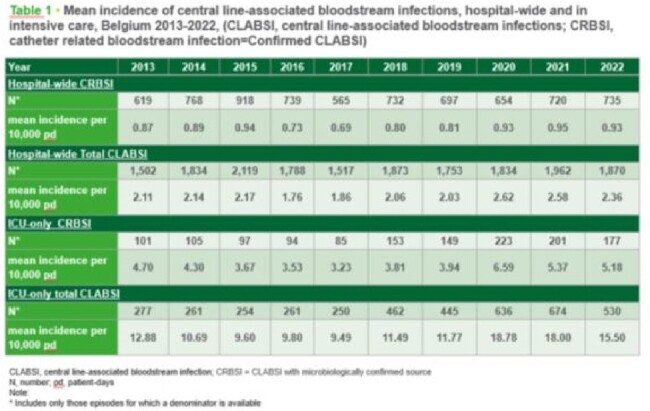

**Methods:**

Data from national surveillance programs were analyzed using negative binomial longitudinal regression models. Incidence changes in CLABSI between pre-pandemic (2013-2019) and 2020-2022 were assessed, along with CDI incidence calculations. Statistical analyses utilized SAS Enterprise Guide 7.13 and STATA 16. Insights into incidence and causative agents for both BSI and CDI were analyzed.

Mean incidence of central line-associated bloodstream infections, hospital-wide,Belgium 2013-2022 ( CLABSI, central line-associated bloodstream infections; CRBSI, catheter related bloodstream infection=Confirmed CLABSI)

**Figure:**
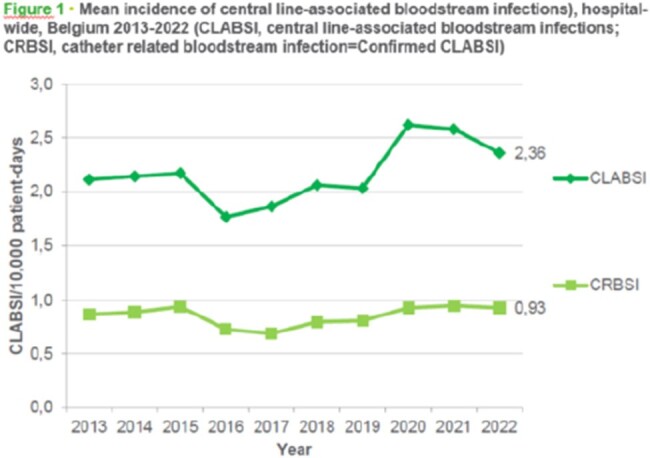

**Results:**

Results show stable BSI incidence from 2014 to 2019. The 2020-2022 incidence calculation included 97, 99, and 95 participating hospitals, respectively. Hospital-wide CLABSI incidence increased from 2.0 to 2.6 per 10,000 patient-days in 2019 and then declined to 2.4 in 2022. ICU-only CLABSI incidence rose from 11.77 to 18.78 in 2020 and declined to 15.50 in 2022. The predominant microorganisms observed in total CLABSI since 2014 were S. epidermidis and E. faecium. In 2022 there was a statistically significant increase of HA-CDI incidence per 10,000 hospital-days compared to 2021, from 1.31 to 1.62. However, even though this increase was observed, most characteristics remain stable, such as percentage of recurrent episodes, age group of patients with infection. Since 2014, the most detected strain remained BR014.

Mean incidence of HA-CDI/10, 000 Hospitalisation-days in hospitals, per region. Belgium, 2010-2022.

**Figure:**
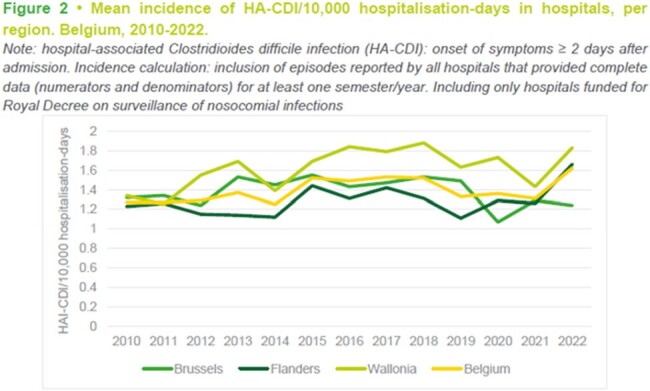

**Conclusion:**

The analysis of BSI and CDI epidemiology reveals significant trends crucial for healthcare management. The impact of the COVID-19 pandemic on CLABSI rates underscores the importance of targeted interventions, while ongoing challenges in HA-CDI incidence highlight the need for continued surveillance improvement. Future efforts should focus on effective prevention and control strategies to mitigate HAIs in Belgian healthcare facilities.

**Disclosures:**

**All Authors**: No reported disclosures

